# A Novel Signature Based on mTORC1 Pathway in Hepatocellular Carcinoma

**DOI:** 10.1155/2020/8291036

**Published:** 2020-09-15

**Authors:** Zhuomao Mo, Shuqiao Zhang, Shijun Zhang

**Affiliations:** ^1^Department of Traditional Chinese Medicine, The First Affiliated Hospital, Sun Yat-Sen University, Guangzhou, Guangdong Province 510080, China; ^2^Hospital of Chengdu University of Traditional Chinese Medicine, Chengdu University of Traditional Chinese Medicine, Chengdu 611137, China

## Abstract

**Background:**

mTORC1 signal pathway plays a role in the initiation and progression of hepatocellular carcinoma (HCC), but no relevant gene signature was developed. This research aimed to explore the potential correlation between the mTORC1 signal pathway and HCC and establish the related gene signature.

**Methods:**

HCC cases were retrieved from The Cancer Genome Atlas (TCGA), International Cancer Genome Consortium (ICGC), and Gene Expression Omnibus (GEO) databases. The genes included in mTORC1-associated signature were selected by performing univariate and multivariate Cox regression analyses and lasso regression analysis. The protein expression level of included genes was verified by The Human Protein Altas. Then, the signature was verified by survival analysis and multiple receiver operating characteristic (ROC) curve. Moreover, the correlation between signature and immune cells infiltration was investigated. Furthermore, a nomogram was established and evaluated by C-index and calibration plot.

**Results:**

The signature was established with the six genes (*ETF1, GSR, SKAP2, HSPD1, CACYBP*, *and PNP*). Three genes (*ETF1*, *GSR*, and *HSPD1*) have verified their protein expression level in HCC. Under the grouping from signature, patients in the high-risk group showed worse survival than those in the low-risk group in both three datasets. The signature was found to be significantly associated with the infiltration of B cells, CD4^+^ T-cells, CD8^+^ T-cells, dendritic cells, macrophages, and neutrophils. The univariate and multivariate Cox regression analysis indicated that mTORC1-related signature could be the potential independent prognostic factor in HCC. Finally, the nomogram involving age, gender, stage, and signature has been established and verified.

**Conclusion:**

The mTORC1-associated gene signature established and validated in our research could be used as a potential prognostic factor in HCC.

## 1. Introduction

Hepatocellular carcinoma (HCC) is one of the most prevalent cancers around the world, becoming the second leading causes of tumor-related death [1]. Owing to the high rate of metastasis, HCC patients with advanced stage are usually with a poor prognosis [2]. Although the treatment and biomarkers of HCC have developed, the clinical outcomes of HCC patients are still unsatisfactory [3]. The occurrent and development of HCC involved interactions between genetics, epigenetics, and transcriptomic alterations [4]. Many studies have verified that different biomarkers have certain prognostic value in HCC. For example, Gu et al. found that *CCL14* was a potential prognostic biomarker that correlated with tumor immune cell infiltration in HCC [5]. Another study found the strong correlations between *PRPF3* expression and prognosis in HCC [6]. However, as a biomarker, a single gene usually has a lower prognostic value than multigene prognostic signature. Therefore, many gene-related signatures have been developed for predicting prognosis of HCC. For instance, Zhang et al. established a gene signature associated with HCC microenvironment and successfully verified it [7]. Other predictive signatures based on immune [8] and glycolysis [9] also play an important role in HCC prognosis.

Generally, the gene researches usually focus on comparing the gene expression between two groups or pay attention to the highly upregulated and downregulated genes. Nevertheless, some genes which showed no significant difference but had important biological function and characteristics were omitted. In view of this, a computational method gene set enrichment analysis (GSEA) determined whether a prior defined set of genes shows statistically significant differences between two biological states [10]. The advantage of GSEA is that it can identify the genes in which expression is based on the trend of overall level. Consequently, in this research, we identified the pathway and gene with GSEA. Then, we constructed the signature based on related genes and verified it, providing the more comprehensive and accurate prognostic model for clinic.

## 2. Materials and Methods

### 2.1. Data Collection

The gene expression data with the type of level 3 RNA-seq FPKM dataset and the clinical messages in TCGA website (https://portal.gdc.cancer.gov/) were retrieved. A total of 377 HCC cases have been downloaded and analyzed. As the validated cohorts, GSE76427 datasets were retrieved from Gene Expression Omnibus (GEO) database (https://www.ncbi.nlm.nih.gov/geo/), while ICGC-LIRI dataset was retrieved from International Cancer Genome Consortium (ICGC) database (https://icgc.org/).

### 2.2. Identification of Pathways and Related Genes

After data collection, we extracted the clinical details and generated the expression matrix of all genes in HCC of TCGA dataset. Then, we divided the patients into two groups according to the survival status and employed GSEA to choose the most relevant pathways. The pathways were considered for further analyses if the normalized *p* value was <0.01. After that, we collected the related genes of involved pathway from Molecular Signatures Database (http://software.broadinstitute.org/gsea/msigdb/index.jsp).

### 2.3. Construction and Verification of Signature

Firstly, we performed the differentially expressed analysis to select the related genes. The “limma” package under R studio software was employed, and a *p* value <0.05 was considered statistically significant. Secondly, we employed the univariate and multivariate Cox regression analysis to choose the prognostic genes among the differentially expressed genes. The genes in this section were eligible for further selection if a *p* value was <0.05. Then, the lasso regression analysis was executed for checking selected genes. In this analysis, a lasso penalty was applied, to simultaneously account for shrinkage and variable selection. The optimal value of the lambda penalty parameter was defined by performing 10 cross-validations. Using the “glmnet” package, the coefficient of each included genes and risk score of each case were calculated. The calculation formula of risk score was as follows:

(1) $$\begin{array}{c} \text{risk score}=\left(\text{coefficient mRNA}1\times \text{expression of mRNA}1\right)+\left(\text{coefficient mRNA}2\times \text{expression of mRNA}2\right)+\cdots +\left(\text{coefficient mRNA}n\times \text{expression mRNA}n\right). \end{array}$$

The cases were divided into two groups (high risk or low risk), according to the risk score median. To explore the time-dependent prognostic value of our gene signature, the survival analysis was performed using the “survival” package in the R studio software. The relationship between signature and other clinical messages was also evaluated and visualized with a heatmap. The protein expression level of included genes of signature was verified by The Human Protein Altas (https://www.proteinatlas.org/). Besides, the multiple receiver operating characteristic (ROC) curve was performed to check the predictive accuracy of risk score. In addition, we investigated the correlation between signature and six different immune cells (B cells, CD4^+^ T-cells, CD8^+^ T-cells, dendritic cells, macrophages, and neutrophils). The infiltration data of six immune cells were retrieved from tumor immune estimation resource (https://cistrome.shinyapps.io/timer/). Moreover, the univariate and multivariate Cox regression analyses were performed to verify whether the risk score is an independent prognostic factor.

### 2.4. Predictive Nomogram Design

A predictive nomogram based on age, gender, stage, and risk score was constructed using the “rms” package and Cox regression model to predict the overall survival (OS) at 1 year, 3 years, and 5 years of HCC patients. Then, we used Harrell's concordance index (C-index) and calibration plot to evaluate the nomogram.

## 3. Results

**Figure 1:**
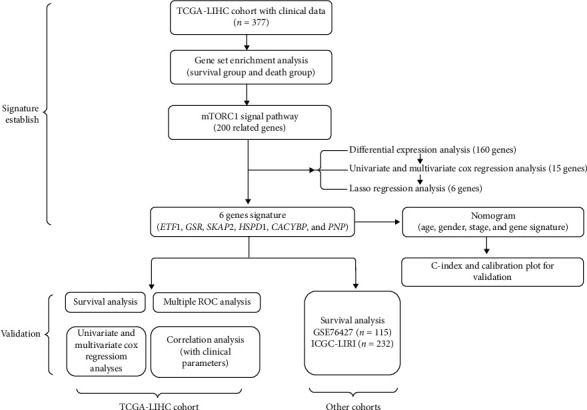
Screening process and validation of this study.

**Figure 2:**
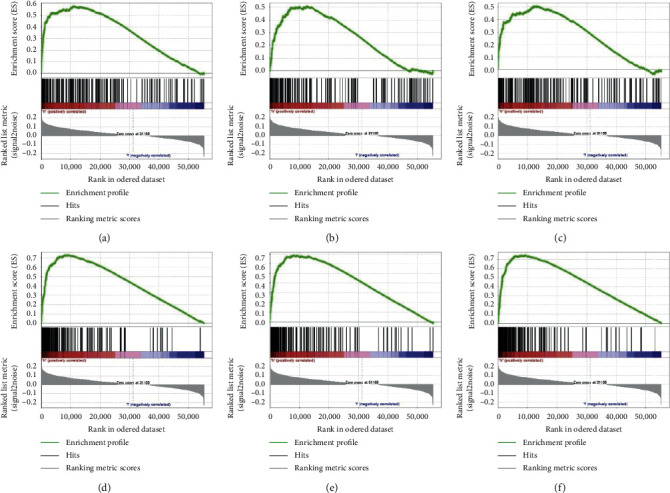
Enrichment plots of signal pathways which importantly differentiated between survival and death groups: (a) HALLMARK_MTORC1_SIGNALLING; (b) HALLMARK_UV_RESPONSE_UP; (c) HALLMARK_GLYCOLYSIS; (d) HALLMARK_G2M_CHECKPOINT; (e) HALLMARK_MYC_TARGETS_V1; (f) HALLMARK_E2F_TARGETS.

**Figure 3:**
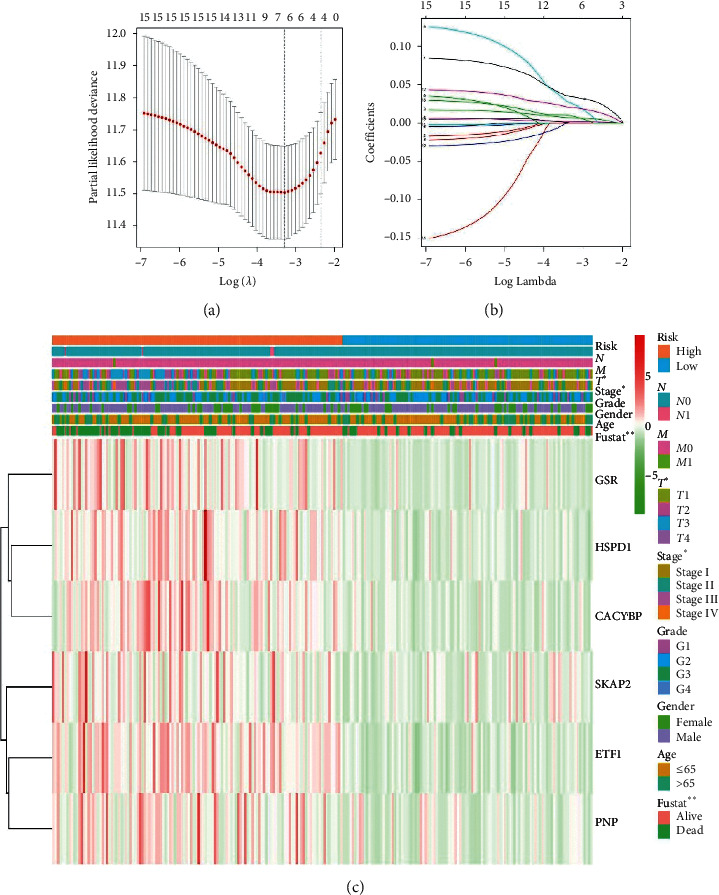
Lasso regression analysis results and heatmap: (a) partial likelihood deviance for the lasso regression; (b) lasso regression analysis; (c) heatmap of signature and clinical parameters.

The clinical data details of the patients used in this study are shown in Table 1.  Figure 1 shows the screening process and validation of our study. The result of GSEA in Figure 2 showed that a total of 6 pathways were eligible, and the details of pathways are summarized in Table 2. Considering the highest normalized enrichment score of mTORC1 pathways, we chose the 200 relevant genes in this pathway for further analysis. A total of 199 genes have been found in gene expression matrix, and the differentially expressed analysis showed that 160 genes were significantly different in HCC (see Supplementary files 1–3). After that, the univariate and multivariate Cox regression analyses demonstrated that 15 genes (*ETF1, CTSC, GSR, HSPE1, SKAP2, HSPD1, TES, TFRC, ASNS, EPRS, CANX, CACYBP, UNG, TBK1, and PNP*) were included with *p* < 0.05 (see Supplementary files 4 and 5). As illustrated in Figures 3(a) and 3(b), the results of lasso regression analysis further confirmed the signature composed of 6 genes (*ETF1, GSR, SKAP2, HSPD1, CACYBP*, *and PNP*). And the coefficients of *ETF1, GSR, SKAP2, HSPD1, CACYBP*, and *PNP* were 0.03402, 0.00670 0.02556, 0.00181, 0.02034, and 0.00916, respectively.

**Figure 4:**
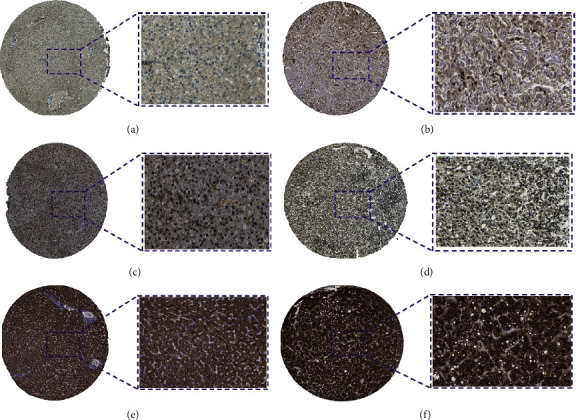
Immunohistochemistry of three included genes: (a) ETF1-normal; (b) ETF1-tumor; (c) GSR-normal; (d) GSR-tumor; (e) HSPD1-normal; (f) HSPD1-tumor.

**Figure 5:**
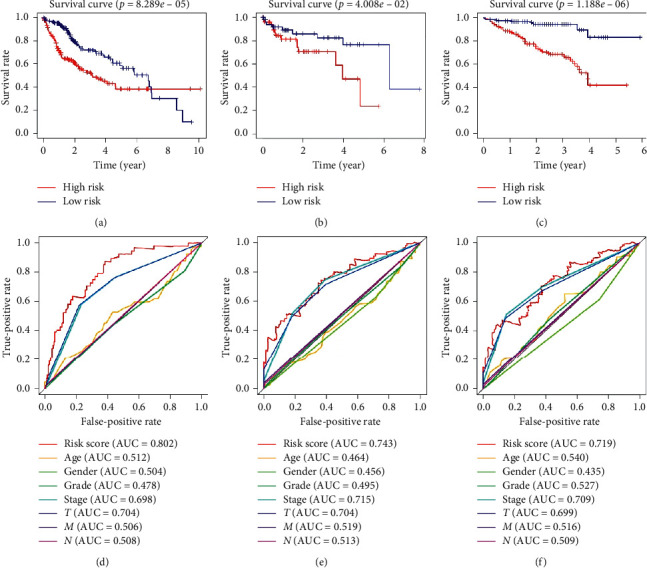
Survival analysis results and multiple ROC results. (a–c) The survival analysis of TCGA-LIHC cohort, GSE76427 cohort, and ICGC-LIRI cohort, respectively. (d–f) Multiple ROC results in 1 year, 3 years, and 5 years, respectively.

**Figure 6:**
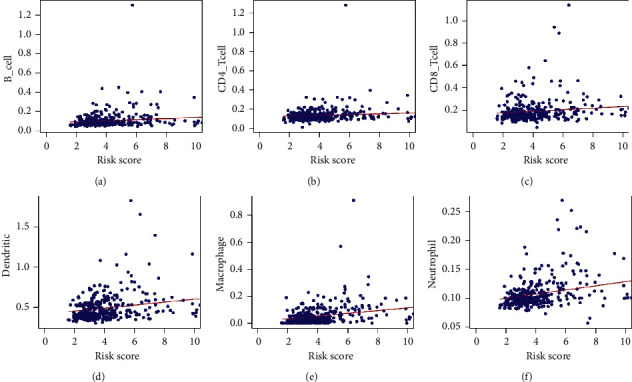
Correlation plot between risk score and immune cells infiltration: (a) Cor = 0.178 (*p*=6.522*e* − 04); (b) Cor = 0.135 (*p*=0.010); (c) Cor = 0.183 (*p*=4.513*e* − 04); (d) Cor = 0.281 (*p*=4.859*e* − 08); (e) Cor = 0.340 (*p*=2.532*e* − 11); (f) Cor = 0.363 (*p*=7.893*e* − 13).

Besides, the heatmap of risk score and clinical parameters was shown in Figure 3(c). All the genes included in the signature were highly expressed in the high-risk group and lowly expressed in the low-risk group. The correlation between gene expression of final included genes and clinical parameters is shown in Table 3. Meanwhile, significant difference was found between risk score and stage, grade, *T*, and *M*, respectively (Table 3). In terms of protein expression, three genes (*ETF1, GSR,* and *HSPD1*) have been found to be highly expressed in HCC tissue according to Figure 4. The survival analysis of TCGA (Figure 5(a)), GSE76427 (Figure 5(b)), and ICGC (Figure 5(c)) both indicated that significant difference was found between two groups (*p* < 0.05). And the multiple ROC curve plot (Figures 5(d)–5(f)) demonstrated that the risk score got the highest predictability among analyzed factors in 1 year (AUC = 0.802), 3 years (AUC = 0.743), and 5 years (AUC = 0.719). In terms of immune cells infiltration, significant difference was found between risk score and B cells, CD4^+^ T-cell, CD8^+^ T-cell, dendrites, macrophage, and neutrophil (Figure 6). Furthermore, we performed the univariate and multivariate Cox regression analyses, and the results in Table 4 revealed that the signature can be the independent prognostic factor in HCC (*p* < 0.01).

**Figure 7:**
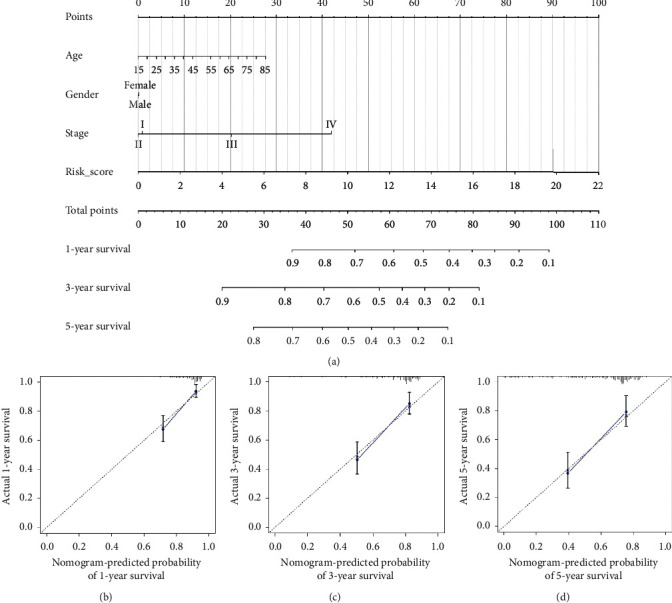
Construction and validation of nomogram: (a) nomogram based on age, gender, stage, and gene signature; (b–d) calibration plot of 1-year, 3-year, and 5-year OS, respectively.

Finally, using the TCGA cohort, we built the nomogram based on age, gender, stage, and established signature to predict 1-year, 3-year, and 5-year OS for HCC patients (Figure 7(a)). The results of decision curve analysis (Supplementary file 6) demonstrated that nomogram with age, gender, stage, and established signature showed a higher benefit than other two solo models (stage only or risk score only). The C-index of nomogram was 0.73, and the calibration plot for the probability of survival at 1 year (Figure 7(b)), 3 years (Figure 6(c)), and 5 years (Figure 6(d)) showed good agreement between the prediction by nomogram and real observation. Also, we established the nomograms and calibration plots based on GEO and ICGC cohorts (Supplementary files 7 and 8), and their C-index was 0.70 and 0.76, respectively.

## 4. Discussion

In the initiation and progression of hepatocellular carcinoma, genetic factors usually play an important role. Meanwhile, mRNA gene signature based on a certain characteristic like glycolysis [11] and immune [12] has been developed for predicting cancer prognosis. In this research, we explored specific function to identify genes by GSEA that could predict the survival of HCC patients. According to our results, six signal pathways were found to be highly related to survival and we established the gene signature with the mTORC1 signal pathway. As we all know, the mTOR pathway is a serine/threonine protein kinase belonging to the PI3K-related kinase family [13], which comprised of two distinct complexes (mTORC1 and mTORC2). With Raptor as its unique and key protein component, mTORC1 plays an important role in cell survival, autophagy, and metabolism [14]. Concerning for the mTOR signal pathway in HCC, it has been found that aberrant mTOR signaling was present in half of the HCC cases [15]. Meanwhile, an intact mTORC1 axis [16] and mTORC2-Akt1 cascade [17] were required for c-Myc-driven hepatocarcinogenesis. Moreover, some research studies [15, 18] provided the theoretical basis of mTOR signaling pathway-oriented targeting treatment for HCC in clinic. Overall, these abovementioned evidences demonstrated that mTOR signal pathway plays an important role in the development of HCC.

In this study, we identified six genes in signature by performing the differentially expressed analysis, univariate Cox regression analysis, and lasso regression analysis. Among our included genes, five genes have been found to be related to HCC from previous studies. Singh et al. found that *ETF1, CNOT6*, and *XRN1* gene in HepG2 cells led to significant alteration in stability of specific mRNAs, and this mechanism may hold novel cancer therapeutic targets [19]. In another research, McLoughlin concluded that *GSR, TRXR1, NRF2*, and oxidative stress determined hepatocellular carcinoma malignancy [20]. Lee's et al. study [21] found that *HSPD1* was downregulated during early apoptosis of the hepatoma cell mediated by Paeoniae Radix. In terms of *CACYBP*, it has been verified that *CACYBP* can promote hepatocellular carcinoma progression in the absence of *RNF41*-mediated degradation [22]. Moreover, a study [23] found that *PNP*/fludarabine suicide gene system induced HCC cell apoptosis and inhibited the growth of HCC cells. Although we found no evidence supporting the correlation between *SKAP2* and HCC, it has been verified that *SKAP2* promotes podosome formation to facilitate tumor-associated macrophage infiltration and metastatic progression [24].

Recently, further investigations have been performed to explore how the mTOR signal transduction mechanisms modulate sensitivity of targeted therapies, angiogenesis, and tumor immunity [25]. The interest in mTOR targeting may improve immune response against cancer and develop new therapeutic strategy. It has been verified that an inflammatory-CCRK circuitry drove mTORC1-dependent metabolic and immunosuppressive reprogramming in obesity-related hepatocellular carcinoma [26]. In another study [27], Tan concluded that Tim-3-mediated PI3K/mTORC1 interference leads to the dysfunction of both tumor-infiltrating conventional natural killer cells and liver-resident natural killer cells. In our research, the results showed that mTORC1 signature significantly associated with B cells, CD4^+^ T-cell, CD8^+^ T-cell, dendrites, macrophage, and neutrophil, which indicated that the patients in high-risk group may benefit from immune-targeted therapies and provide a new strategy for immune checkpoint-based targeting.

Being different from the previous prognostic studies in HCC, our predictive model firstly concentrated on the mTORC1 signal pathway. More importantly, the mTORC1 signal pathway was identified by GSEA, which indicated the underlying mechanism between survival of HCC and mTORC1 pathway. Moreover, the validation from three independent datasets and a rigorous screening process enabled the identification of a reliable signature. However, our study has some limitations. First, prognostic signature showed a relatively low diagnostic performance in predicting 5-year OS. It may be attributed to that only 200 associated genes were defined and evaluated for the initiation of the screening process. Second, using a single characteristic (mTORC1 signal pathway) to establish the predictive model is an intrinsic weakness. Indeed, many other mechanisms, such as metabolism [28] and immune [8], have an influence on the development and progression of HCC. Third, the weak correlation between risk score and immune cells infiltration was observed in our research, which may attribute to the calculated method (TIMER) of immune cell infiltration. Furthermore, our signature explored the underlying effect between mTOR signal pathway and HCC, but it is necessary to perform more independent trials and functional experiments to shed light on the mechanism linking them.

## 5. Conclusion

Our study is the first to identify a novel gene signature related to mTORC1 signal pathway that could be used as a potential prognostic factor in hepatocellular carcinoma.

## Figures and Tables

**Table 1:** Baseline patient characteristic in TCGA, GEO, and ICGC cohorts.

Clinical characteristics		Number	Percent (%)
*TCGA-LIHC (n* *=* *377)*			
Survival status	Survival	249	66
	Death	128	34
Age (1 patient missing)	≤65 years	235	62.5
	>65 years	141	37.5
Gender	Female	122	68
	Male	255	32
Stage (24 patients missing)	I	175	50
	II	87	24.6
	III	86	24.4
	IV	5	1
Grade (5 patients missing)	G1	55	14
	G2	180	48
	G3	124	33
	G4	13	5
T classification (3 patients missing)	T1	185	49
	T2	95	26
	T3	81	22
	T4	13	3

*GSE76427 (n* *=* *115)*			
Survival status	Survival	92	80
	Death	23	20
Age	≤65 years	65	56.5
	>65 years	50	43.5
Gender	Female	22	19.1
	Male	93	80.9
Stage	I	55	47.8
	II	35	30.4
	III	21	18.3
	IV	4	3.5

*ICGC-LIRI (n* *=* *260)*			
Survival status	Survival	214	82.4
	Death	46	17.6
Age	≤65 years	98	37.7
	>65 years	162	62.3
Stage	I	40	15.4
	II	117	45
	III	80	30.8
	IV	23	8.8

**Table 2:** Details of signal pathways selected by GSEA.

Name	Size	ES	NES	Normalized *p* value
MTORC1 SIGNALING	200	0.581694	1.982214	0
UV_RESPONSE_UP	158	0.506378	1.955258	0
GLYCOLYSIS	200	0.506672	1.88337	0
G2M_CHECKPOINT	199	0.73077	1.881815	0.002088
MYC_TARGETS_V1	199	0.685811	1.860498	0.004141
E2F_TARGETS	200	0.745006	1.856894	0.002079

ES = enrichment score; NES = normalized enrichment score.

**Table 3:** Correlation between included genes (signature) and clinical parameters.

Id	Age	Gender	Grade	Stage	*T*	*M*	*N*
ETF1	0.191 (0.849)	0.209 (0.834)	−1.899 (0.059)	−1.797 (0.075)	−1.298 (0.197)	0.547 (0.636)	−1.291 (0.285)
GSR	0.782 (0.436)	−1.263 (0.208)	**−2.216 (0.028)**	−1.886 (0.062)	**−2.008 (0.047)**	2.607 (0.093)	**2.35 (0.049)**
SKAP2	−0.721 (0.473)	1.488 (0.139)	1.274 (0.204)	**−2.457 (0.016)**	**−2.009 (0.047)**	1.576 (0.241)	−0.815 (0.474)
HSPD1	0.988 (0.325)	−0.442 (0.659)	**−2.609 (0.010)**	−1.418 (0.158)	−1.17 (0.244)	3.016 (0.067)	−1.426 (0.244)
CACYBP	**2.79 (0.006)**	−0.636 (0.526)	**−2.635 (0.009)**	−1.547 (0.125)	−1.71 (0.090)	0.38 (0.738)	−0.015 (0.989)
PNP	1.614 (0.109)	1.766 (0.080)	−1.139 (0.256)	**−2.781 (0.006)**	**−2.819 (0.006)**	1.759 (0.202)	−0.813 (0.475)
Risk score	0.94 (0.054)	−0.45 (0.654)	**−2.687 (0.008)**	**−2.603 (0.010)**	**−2.428 (0.017)**	**4.473 (0.009)**	−1.171 (0.317)

The results in table represent correlation coefficient and *p* value; the bold represents significantly different values.

**Table 4:** Results of univariate and multivariate Cox regression analyses.

	*p* value	Hazard ratio
*Univariate Cox regression analysis*		
Age	0.591	1.005 (0.987, 1.023)
Gender	0.301	0.780 (0.487, 1.249)
Grade	0.914	1.017 (0.746, 1.387)
Stage	<0.001	1.865 (1.456, 2.388)
*T*	<0.001	1.804 (1.434, 2.270)
*M*	0.023	3.850 (1.207, 12.281)
*N*	0.328	2.022 (0.494, 8.276)
Risk score	<0.001	1.205 (1.142, 1.272)

*Multivariate Cox regression analysis*		
Age	0.064	1.019 (0.999, 1.040)
Gender	0.870	0.957 (0.567, 1.617)
Grade	0.707	0.935 (0.659, 1.327)
Stage	0.896	0.935 (0.340, 2.569)
*T*	0.223	1.752 (0.711, 4.314)
*M*	0.317	1.990 (0.518, 7.654)
*N*	0.595	1.686 (0.246, 11.564)
Risk score	<0.001	1.236 (1.152, 1.328)

## Data Availability

The datasets generated and/or analyzed during the current study are available in the TCGA (https://portal.gdc.cancer.gov/), GEO (https://www.ncbi.nlm.nih.gov/geo/), and ICGC (https://icgc.org/).

## Abbreviations

HCC: Hepatocellular carcinoma
TCGA: The Cancer Genome Atlas
ICGC: International Cancer Genome Consortium
GEO: Gene Expression Omnibus
ROC: Receiver operating characteristic
GSEA: Gene set enrichment analysis
OS: Overall survival
C-index: Concordance index.

## Supplementary Materials

Supplementary Materials Supplementary file 1: the gene expression matrix of 199 mTORC1-related genes (TXT 890 kb). Supplementary file 2: the heatmap of 199 mTORC1-related genes (JPG 10489 kb). Supplementary file 3: the volcano plot of 199 mTORC1-related genes (JPG 571 kb). Supplementary file 4: the results of univariate Cox regression analysis for 160 genes (DOC 26.2 kb). Supplementary file 5: the results of multivariate Cox regression analysis for 101 genes (DOC 22.7 kb). Supplementary file 6: the results of decision curve analysis for three different models (JPG 988 kb). Supplementary file 7: the nomogram and calibration plots based on GEO cohort (JPG 608 kb). Supplementary file 8: the nomogram and calibration plots based on ICGC cohort (JPG 653 kb).
