# Supplementary material for: A Novel Signature Based on mTORC1 Pathway in Hepatocellular Carcinoma
Source: J Oncol. 2020 Sep 15;2020:8291036. doi: 10.1155/2020/8291036 (PMC7512110; doi:10.1155/2020/8291036)
Supplement: Supplementary Materials — Supplementary file 1: the gene expression matrix of 199 mTORC1-related genes (TXT 890 kb). Supplementary file 2: the heatmap of 199 mTORC1-related genes (JPG 10489 kb). Supplementary file 3: the volcano plot of 199 mTORC1-related genes (JPG 571 kb). Supplementary file 4: the results of univariate Cox regression analysis for 160 genes (DOC 26.2 kb). Supplementary file 5: the results of multivariate Cox regression analysis for 101 genes (DOC 22.7 kb). Supplementary file 6: the results of decision curve analysis for three different models (JPG 988 kb). Supplementary file 7: the nomogram and calibration plots based on GEO cohort (JPG 608 kb). Supplementary file 8: the nomogram and calibration plots based on ICGC cohort (JPG 653 kb). [file 8291036.f1.zip › 8291036.f1/Supplementary file 4.docx]

| id | HR | HR.95L | HR.95H | p-value |
| --- | --- | --- | --- | --- |
| TXNRD1 | 1.014303 | 1.009868 | 1.018757 | 2.11E-10 |
| G6PD | 1.012598 | 1.008443 | 1.01677 | 2.40E-09 |
| STIP1 | 1.030891 | 1.020564 | 1.041323 | 3.18E-09 |
| CCT6A | 1.027875 | 1.018545 | 1.037291 | 3.44E-09 |
| SQSTM1 | 1.003119 | 1.002079 | 1.004159 | 3.96E-09 |
| ENO1 | 1.002016 | 1.001315 | 1.002719 | 1.76E-08 |
| PLK1 | 1.126269 | 1.080095 | 1.174418 | 2.59E-08 |
| NUP205 | 1.210735 | 1.131318 | 1.295726 | 3.31E-08 |
| PLOD2 | 1.042063 | 1.026903 | 1.057446 | 3.57E-08 |
| PSMD14 | 1.101577 | 1.064196 | 1.14027 | 3.97E-08 |
| ABCF2 | 1.114563 | 1.071259 | 1.159617 | 8.12E-08 |
| PRDX1 | 1.003033 | 1.001905 | 1.004162 | 1.33E-07 |
| TUBG1 | 1.027891 | 1.017388 | 1.038503 | 1.52E-07 |
| ETF1 | 1.07503 | 1.046149 | 1.104708 | 1.92E-07 |
| GTF2H1 | 1.278441 | 1.163879 | 1.404279 | 2.93E-07 |
| CACYBP | 1.05061 | 1.030802 | 1.070799 | 3.70E-07 |
| BUB1 | 1.259142 | 1.151876 | 1.376396 | 3.93E-07 |
| GMPS | 1.159266 | 1.094462 | 1.227906 | 4.77E-07 |
| RRM2 | 1.054048 | 1.032308 | 1.076246 | 7.41E-07 |
| RIT1 | 1.172479 | 1.100832 | 1.248788 | 7.57E-07 |
| CCNF | 1.347079 | 1.196502 | 1.516606 | 8.38E-07 |
| HSPD1 | 1.004804 | 1.002884 | 1.006728 | 9.09E-07 |
| MCM2 | 1.047985 | 1.028327 | 1.068019 | 1.23E-06 |
| PNO1 | 1.135671 | 1.078665 | 1.195689 | 1.29E-06 |
| GSR | 1.019132 | 1.011159 | 1.027168 | 2.25E-06 |
| MCM4 | 1.0617 | 1.035352 | 1.08872 | 3.02E-06 |
| SLC2A1 | 1.061942 | 1.03534 | 1.089228 | 3.43E-06 |
| POLR3G | 2.158132 | 1.553854 | 2.997407 | 4.44E-06 |
| HSPA4 | 1.039836 | 1.022559 | 1.057405 | 4.89E-06 |
| ME1 | 1.031504 | 1.017389 | 1.045814 | 1.02E-05 |
| PPIA | 1.011102 | 1.005813 | 1.01642 | 3.70E-05 |
| CDC25A | 1.238658 | 1.117247 | 1.373264 | 4.78E-05 |
| TUBA4A | 1.022263 | 1.011268 | 1.033379 | 6.59E-05 |
| PSME3 | 1.047759 | 1.023647 | 1.07244 | 8.59E-05 |
| EIF2S2 | 1.026657 | 1.013249 | 1.040243 | 8.77E-05 |
| RRP9 | 1.04267 | 1.020757 | 1.065053 | 0.000115 |
| SLC1A5 | 1.01226 | 1.005874 | 1.018687 | 0.000161 |
| ACLY | 1.027068 | 1.012826 | 1.04151 | 0.000178 |
| ACTR3 | 1.07173 | 1.033233 | 1.111661 | 0.000206 |
| ACSL3 | 1.041296 | 1.019148 | 1.063925 | 0.000225 |
| GAPDH | 1.000639 | 1.000292 | 1.000985 | 0.000302 |
| ACACA | 1.122547 | 1.053769 | 1.195815 | 0.000339 |
| PSMA3 | 1.036802 | 1.01599 | 1.058041 | 0.000477 |
| GLA | 1.027138 | 1.011764 | 1.042745 | 0.000502 |
| UNG | 1.037021 | 1.015951 | 1.058528 | 0.000519 |
| SLC9A3R1 | 1.003819 | 1.001658 | 1.005984 | 0.000527 |
| RPN1 | 1.010225 | 1.004427 | 1.016057 | 0.000532 |
| SKAP2 | 1.10964 | 1.045571 | 1.177635 | 0.000607 |
| IFI30 | 1.956193 | 1.331779 | 2.873368 | 0.000625 |
| SLC7A11 | 1.098522 | 1.03947 | 1.160928 | 0.000859 |
| PGK1 | 1.005307 | 1.002167 | 1.008457 | 0.000912 |
| EEF1E1 | 1.090515 | 1.035681 | 1.148252 | 0.000995 |
| GSK3B | 1.154024 | 1.058767 | 1.25785 | 0.001118 |
| NFYC | 1.095495 | 1.036371 | 1.157991 | 0.001273 |
| PSMD12 | 1.068931 | 1.026063 | 1.11359 | 0.001413 |
| PSMB5 | 1.012807 | 1.00475 | 1.020929 | 0.001792 |
| ACTR2 | 1.01685 | 1.006231 | 1.027582 | 0.001811 |
| PNP | 1.050395 | 1.018399 | 1.083397 | 0.001839 |
| M6PR | 1.051208 | 1.018542 | 1.084922 | 0.001931 |
| PSMG1 | 1.074938 | 1.025883 | 1.126338 | 0.002427 |
| YKT6 | 1.019744 | 1.006914 | 1.032737 | 0.002473 |
| SORD | 0.986233 | 0.977227 | 0.995323 | 0.003062 |
| PSPH | 1.027065 | 1.008968 | 1.045487 | 0.003238 |
| IMMT | 1.027831 | 1.009104 | 1.046906 | 0.003434 |
| PSMC6 | 1.13051 | 1.041308 | 1.227354 | 0.003442 |
| ATP6V1D | 1.069091 | 1.022247 | 1.118081 | 0.003473 |
| TPI1 | 1.002564 | 1.000795 | 1.004335 | 0.004479 |
| EGLN3 | 1.049863 | 1.015104 | 1.085812 | 0.004616 |
| ALDOA | 1.002487 | 1.000745 | 1.004232 | 0.005128 |
| RPA1 | 1.05668 | 1.016555 | 1.098388 | 0.00525 |
| PSMD13 | 1.023256 | 1.006764 | 1.040019 | 0.005553 |
| SLC1A4 | 1.058174 | 1.015695 | 1.102429 | 0.006832 |
| HMBS | 1.085231 | 1.021726 | 1.152682 | 0.007847 |
| HSPE1 | 1.004799 | 1.001203 | 1.008407 | 0.008855 |
| ASNS | 1.052759 | 1.01226 | 1.094878 | 0.010205 |
| CTSC | 1.022576 | 1.005193 | 1.040261 | 0.01071 |
| BCAT1 | 1.067245 | 1.015086 | 1.122085 | 0.010908 |
| EPRS | 1.02084 | 1.004652 | 1.037288 | 0.011436 |
| TBK1 | 1.137591 | 1.028294 | 1.258506 | 0.012373 |
| ARPC5L | 1.043697 | 1.009018 | 1.079568 | 0.013114 |
| SSR1 | 1.028475 | 1.005505 | 1.05197 | 0.014838 |
| AURKA | 1.022575 | 1.004367 | 1.041113 | 0.01488 |
| COPS5 | 1.061557 | 1.011382 | 1.114222 | 0.015602 |
| RAB1A | 1.011895 | 1.002234 | 1.021649 | 0.015696 |
| SQLE | 1.008712 | 1.001525 | 1.01595 | 0.017425 |
| TFRC | 1.020786 | 1.002944 | 1.038944 | 0.022209 |
| PDAP1 | 1.010416 | 1.001364 | 1.01955 | 0.024021 |
| HSPA9 | 1.005222 | 1.000442 | 1.010025 | 0.032228 |
| PSMC4 | 1.008824 | 1.000692 | 1.017022 | 0.033385 |
| TES | 1.023096 | 1.001575 | 1.04508 | 0.035287 |
| CANX | 1.002791 | 1.000129 | 1.00546 | 0.039869 |
| QDPR | 0.989318 | 0.979169 | 0.999572 | 0.041224 |
| NMT1 | 1.027856 | 1.00074 | 1.055707 | 0.043987 |
| PHGDH | 1.010444 | 1.000039 | 1.020956 | 0.049146 |
| HPRT1 | 1.014193 | 0.999917 | 1.028673 | 0.051351 |
| DDX39A | 1.016046 | 0.999813 | 1.032543 | 0.052727 |
| DHCR24 | 1.001175 | 0.999981 | 1.002371 | 0.053784 |
| ERO1A | 1.016763 | 0.999316 | 1.034514 | 0.059766 |
| TMEM97 | 1.007 | 0.999493 | 1.014563 | 0.067669 |
| TRIB3 | 1.00501 | 0.999589 | 1.010461 | 0.070152 |
| PDK1 | 1.089718 | 0.992629 | 1.196304 | 0.071142 |
| PSMC2 | 1.017278 | 0.998446 | 1.036465 | 0.072355 |
| IDI1 | 1.004966 | 0.999414 | 1.010549 | 0.07966 |
| PPA1 | 1.015584 | 0.997433 | 1.034066 | 0.092829 |
| LDLR | 0.979122 | 0.955221 | 1.003621 | 0.094263 |
| SERPINH1 | 1.003714 | 0.999271 | 1.008177 | 0.101453 |
| NUPR1 | 1.002433 | 0.999405 | 1.00547 | 0.115389 |
| UCHL5 | 1.047138 | 0.988048 | 1.109761 | 0.120127 |
| SERP1 | 1.013491 | 0.996498 | 1.030774 | 0.120355 |
| CYB5B | 1.022561 | 0.993647 | 1.052317 | 0.127384 |
| DDIT4 | 1.003894 | 0.998754 | 1.00906 | 0.137847 |
| PSAT1 | 1.003575 | 0.998805 | 1.008367 | 0.142108 |
| MTHFD2 | 1.069251 | 0.975116 | 1.172474 | 0.154436 |
| PSMA4 | 1.024183 | 0.990966 | 1.058514 | 0.155464 |
| MLLT11 | 0.960408 | 0.907128 | 1.016816 | 0.165354 |
| TM7SF2 | 0.996847 | 0.992352 | 1.001361 | 0.170716 |
| IFRD1 | 1.038492 | 0.981274 | 1.099047 | 0.191484 |
| SRD5A1 | 0.977172 | 0.943359 | 1.012197 | 0.198712 |
| DHCR7 | 1.002215 | 0.998543 | 1.0059 | 0.237499 |
| ATP2A2 | 1.011879 | 0.991956 | 1.032202 | 0.244453 |
| PPP1R15A | 1.008265 | 0.994289 | 1.022438 | 0.247774 |
| HSPA5 | 1.000881 | 0.999353 | 1.002411 | 0.258783 |
| NUFIP1 | 1.155246 | 0.895072 | 1.491045 | 0.267648 |
| WARS | 1.005459 | 0.995697 | 1.015315 | 0.274096 |
| PGM1 | 0.994621 | 0.984897 | 1.004441 | 0.281916 |
| TCEA1 | 1.013742 | 0.988259 | 1.039881 | 0.293378 |
| BHLHE40 | 1.00261 | 0.997364 | 1.007883 | 0.330133 |
| SDF2L1 | 1.002604 | 0.997328 | 1.007907 | 0.334001 |
| NFKBIB | 1.013122 | 0.986362 | 1.040609 | 0.339807 |
| PITPNB | 1.021482 | 0.977804 | 1.06711 | 0.340463 |
| UFM1 | 1.018874 | 0.979618 | 1.059703 | 0.35096 |
| LTA4H | 1.023228 | 0.973117 | 1.075919 | 0.370095 |
| GOT1 | 0.999033 | 0.996895 | 1.001177 | 0.376445 |
| STC1 | 1.018878 | 0.976811 | 1.062757 | 0.384645 |
| ATP5MC1 | 1.002103 | 0.997059 | 1.007173 | 0.414516 |
| TOMM40 | 1.002908 | 0.99588 | 1.009985 | 0.418308 |
| CCNG1 | 1.004382 | 0.993586 | 1.015294 | 0.427786 |
| HSP90B1 | 1.000584 | 0.999132 | 1.002038 | 0.430475 |
| MAP2K3 | 1.006268 | 0.988669 | 1.02418 | 0.487644 |
| DDIT3 | 1.001959 | 0.995967 | 1.007987 | 0.52251 |
| PIK3R3 | 0.965936 | 0.863383 | 1.080671 | 0.545047 |
| FGL2 | 1.011137 | 0.970025 | 1.053992 | 0.601008 |
| HMGCR | 1.005952 | 0.983472 | 1.028946 | 0.60681 |
| NFIL3 | 0.996904 | 0.984143 | 1.009832 | 0.637177 |
| MTHFD2L | 1.03346 | 0.895942 | 1.192087 | 0.651443 |
| CFP | 0.971961 | 0.831665 | 1.135925 | 0.720669 |
| FDXR | 0.994549 | 0.965073 | 1.024925 | 0.721767 |
| GGA2 | 1.00743 | 0.961917 | 1.055097 | 0.753637 |
| NAMPT | 0.998201 | 0.986642 | 1.009895 | 0.761828 |
| SC5D | 0.998319 | 0.987394 | 1.009365 | 0.764416 |
| GPI | 1.000906 | 0.994916 | 1.006932 | 0.76741 |
| SEC11A | 1.003332 | 0.977236 | 1.030124 | 0.804605 |
| FAM129A | 0.995082 | 0.94753 | 1.04502 | 0.843561 |
| FADS1 | 0.999442 | 0.993547 | 1.005372 | 0.853298 |
| ELOVL5 | 1.000916 | 0.99092 | 1.011013 | 0.858103 |
| CALR | 1.000059 | 0.999305 | 1.000814 | 0.877508 |
| FKBP2 | 1.000257 | 0.995748 | 1.004787 | 0.91108 |
| CTH | 1.000108 | 0.997649 | 1.002572 | 0.931696 |
| PFKL | 1.000763 | 0.978236 | 1.023808 | 0.947678 |
| HMGCS1 | 1.000148 | 0.994982 | 1.00534 | 0.955465 |
