# Supplementary material for: A Novel Signature Based on mTORC1 Pathway in Hepatocellular Carcinoma
Source: J Oncol. 2020 Sep 15;2020:8291036. doi: 10.1155/2020/8291036 (PMC7512110; doi:10.1155/2020/8291036)
Supplement: Supplementary Materials — Supplementary file 1: the gene expression matrix of 199 mTORC1-related genes (TXT 890 kb). Supplementary file 2: the heatmap of 199 mTORC1-related genes (JPG 10489 kb). Supplementary file 3: the volcano plot of 199 mTORC1-related genes (JPG 571 kb). Supplementary file 4: the results of univariate Cox regression analysis for 160 genes (DOC 26.2 kb). Supplementary file 5: the results of multivariate Cox regression analysis for 101 genes (DOC 22.7 kb). Supplementary file 6: the results of decision curve analysis for three different models (JPG 988 kb). Supplementary file 7: the nomogram and calibration plots based on GEO cohort (JPG 608 kb). Supplementary file 8: the nomogram and calibration plots based on ICGC cohort (JPG 653 kb). [file 8291036.f1.zip › 8291036.f1/Supplementary file 5.docx]

| id | HR | HR.95L | HR.95H | pvalue |
| --- | --- | --- | --- | --- |
| ETF1 | 1.207221 | 1.08771 | 1.339864 | 0.000399 |
| CTSC | 0.902081 | 0.844398 | 0.963704 | 0.002239 |
| GSR | 1.033628 | 1.010033 | 1.057774 | 0.004995 |
| HSPE1 | 0.9795 | 0.965232 | 0.993978 | 0.005662 |
| SKAP2 | 1.237497 | 1.062217 | 1.441702 | 0.006247 |
| HSPD1 | 1.013985 | 1.002876 | 1.025218 | 0.013477 |
| TES | 0.898157 | 0.823586 | 0.979479 | 0.015147 |
| TFRC | 0.941208 | 0.894001 | 0.990908 | 0.021008 |
| ASNS | 1.114024 | 1.014195 | 1.223681 | 0.024183 |
| EPRS | 0.9486 | 0.905689 | 0.993544 | 0.02547 |
| CANX | 0.99001 | 0.981265 | 0.998833 | 0.026562 |
| CACYBP | 1.07172 | 1.007669 | 1.139842 | 0.027597 |
| UNG | 1.065929 | 1.002976 | 1.132832 | 0.039817 |
| TBK1 | 0.707021 | 0.507952 | 0.984106 | 0.039886 |
| PNP | 1.073353 | 1.001795 | 1.150022 | 0.044334 |
| SORD | 0.981953 | 0.964216 | 1.000015 | 0.050193 |
| PHGDH | 0.974083 | 0.948531 | 1.000323 | 0.052849 |
| ENO1 | 1.002995 | 0.999892 | 1.006108 | 0.058547 |
| AURKA | 1.06126 | 0.997736 | 1.128828 | 0.059028 |
| ACSL3 | 0.948529 | 0.897834 | 1.002086 | 0.059349 |
| ACLY | 0.957162 | 0.912739 | 1.003746 | 0.070958 |
| PSMA3 | 1.051527 | 0.994594 | 1.11172 | 0.076878 |
| TPI1 | 1.007429 | 0.998991 | 1.015937 | 0.084575 |
| RAB1A | 0.974506 | 0.945805 | 1.004077 | 0.090415 |
| PSMD14 | 1.158802 | 0.976543 | 1.375077 | 0.091393 |
| TUBA4A | 1.024429 | 0.99508 | 1.054644 | 0.103656 |
| PGK1 | 0.989405 | 0.976678 | 1.002297 | 0.106825 |
| PSMG1 | 0.906359 | 0.801286 | 1.025211 | 0.117838 |
| BUB1 | 0.69753 | 0.433521 | 1.122317 | 0.137698 |
| ALDOA | 0.994333 | 0.98652 | 1.002208 | 0.157965 |
| PLOD2 | 1.034429 | 0.98324 | 1.088282 | 0.191136 |
| EEF1E1 | 1.146903 | 0.931837 | 1.411605 | 0.195787 |
| RIT1 | 1.144847 | 0.923148 | 1.419788 | 0.218029 |
| GSK3B | 1.160682 | 0.913551 | 1.474666 | 0.222539 |
| ARPC5L | 0.940639 | 0.845329 | 1.046696 | 0.261569 |
| ACACA | 1.117437 | 0.906683 | 1.377179 | 0.297741 |
| CCT6A | 0.975457 | 0.930544 | 1.022537 | 0.301489 |
| TUBG1 | 1.028652 | 0.974166 | 1.086186 | 0.308984 |
| GMPS | 0.897198 | 0.725488 | 1.109549 | 0.316898 |
| RPN1 | 1.007478 | 0.99285 | 1.022323 | 0.318098 |
| SQLE | 1.008796 | 0.991491 | 1.026402 | 0.321224 |
| SLC9A3R1 | 1.002926 | 0.996855 | 1.009034 | 0.345644 |
| ATP6V1D | 0.948672 | 0.849778 | 1.059075 | 0.348188 |
| NUP205 | 1.150154 | 0.838251 | 1.578112 | 0.386064 |
| COPS5 | 1.052393 | 0.937011 | 1.181983 | 0.388747 |
| SLC1A5 | 1.01318 | 0.981404 | 1.045985 | 0.420598 |
| M6PR | 0.957808 | 0.859825 | 1.066957 | 0.433681 |
| GLA | 1.017713 | 0.973473 | 1.063964 | 0.438741 |
| PSPH | 1.01908 | 0.967979 | 1.072879 | 0.471477 |
| IFI30 | 1.434961 | 0.530762 | 3.879545 | 0.476666 |
| ME1 | 1.010941 | 0.980311 | 1.042529 | 0.48818 |
| QDPR | 0.993425 | 0.974917 | 1.012284 | 0.491744 |
| PPIA | 1.00695 | 0.987036 | 1.027265 | 0.49678 |
| SSR1 | 1.023503 | 0.956588 | 1.095099 | 0.500684 |
| CDC25A | 0.849463 | 0.525775 | 1.372425 | 0.505052 |
| NMT1 | 0.971602 | 0.889923 | 1.060778 | 0.52021 |
| MCM4 | 0.963524 | 0.858174 | 1.081808 | 0.52938 |
| PSMC6 | 1.069707 | 0.865951 | 1.321406 | 0.531964 |
| RRM2 | 0.975663 | 0.90112 | 1.056373 | 0.543474 |
| NFYC | 0.945584 | 0.789299 | 1.132813 | 0.543826 |
| CCNF | 0.863342 | 0.532495 | 1.399748 | 0.55118 |
| G6PD | 0.995066 | 0.978409 | 1.012007 | 0.565809 |
| POLR3G | 1.237071 | 0.587525 | 2.604729 | 0.575471 |
| HSPA4 | 0.986378 | 0.939806 | 1.035259 | 0.57836 |
| ACTR2 | 1.016797 | 0.958022 | 1.079178 | 0.583468 |
| PSMC4 | 0.993731 | 0.970831 | 1.017171 | 0.59702 |
| IMMT | 1.018302 | 0.950748 | 1.090656 | 0.604563 |
| SQSTM1 | 1.000847 | 0.997279 | 1.004427 | 0.642251 |
| PSMD13 | 0.987686 | 0.932919 | 1.045667 | 0.670317 |
| GTF2H1 | 1.083358 | 0.747807 | 1.569476 | 0.672039 |
| PSME3 | 1.021986 | 0.916222 | 1.139958 | 0.696406 |
| SLC7A11 | 1.027759 | 0.88914 | 1.187988 | 0.71108 |
| EGLN3 | 1.024532 | 0.894479 | 1.173493 | 0.726402 |
| GAPDH | 0.999748 | 0.998295 | 1.001203 | 0.733844 |
| ACTR3 | 0.965849 | 0.789879 | 1.181022 | 0.734903 |
| PRDX1 | 0.999409 | 0.995879 | 1.002953 | 0.743505 |
| PDAP1 | 1.006602 | 0.966681 | 1.048173 | 0.749935 |
| HMBS | 0.977841 | 0.849337 | 1.125789 | 0.755256 |
| MCM2 | 0.983245 | 0.883661 | 1.094053 | 0.756465 |
| ABCF2 | 1.026094 | 0.867483 | 1.213707 | 0.763667 |
| HSPA9 | 0.997795 | 0.981803 | 1.014048 | 0.788912 |
| PSMB5 | 0.997287 | 0.977468 | 1.017509 | 0.790843 |
| STIP1 | 0.993248 | 0.942192 | 1.047071 | 0.80133 |
| PNO1 | 0.971724 | 0.770311 | 1.225802 | 0.808759 |
| SLC1A4 | 0.989113 | 0.883713 | 1.107084 | 0.848985 |
| PLK1 | 1.02314 | 0.782396 | 1.33796 | 0.867267 |
| RPA1 | 0.993095 | 0.871289 | 1.13193 | 0.917342 |
| PSMD12 | 0.994812 | 0.875139 | 1.130849 | 0.936599 |
| RRP9 | 0.997452 | 0.922107 | 1.078954 | 0.949245 |
| YKT6 | 0.998056 | 0.937933 | 1.062034 | 0.951062 |
| EIF2S2 | 0.999066 | 0.95135 | 1.049176 | 0.970155 |
| BCAT1 | 0.996304 | 0.819683 | 1.210983 | 0.970335 |
| SLC2A1 | 1.001198 | 0.910581 | 1.100832 | 0.980271 |
| TXNRD1 | 0.999838 | 0.984316 | 1.015606 | 0.983837 |
