# Supplementary figures and images for: A Novel Signature Based on mTORC1 Pathway in Hepatocellular Carcinoma
Source: J Oncol. 2020 Sep 15;2020:8291036. doi: 10.1155/2020/8291036 (PMC7512110; doi:10.1155/2020/8291036)

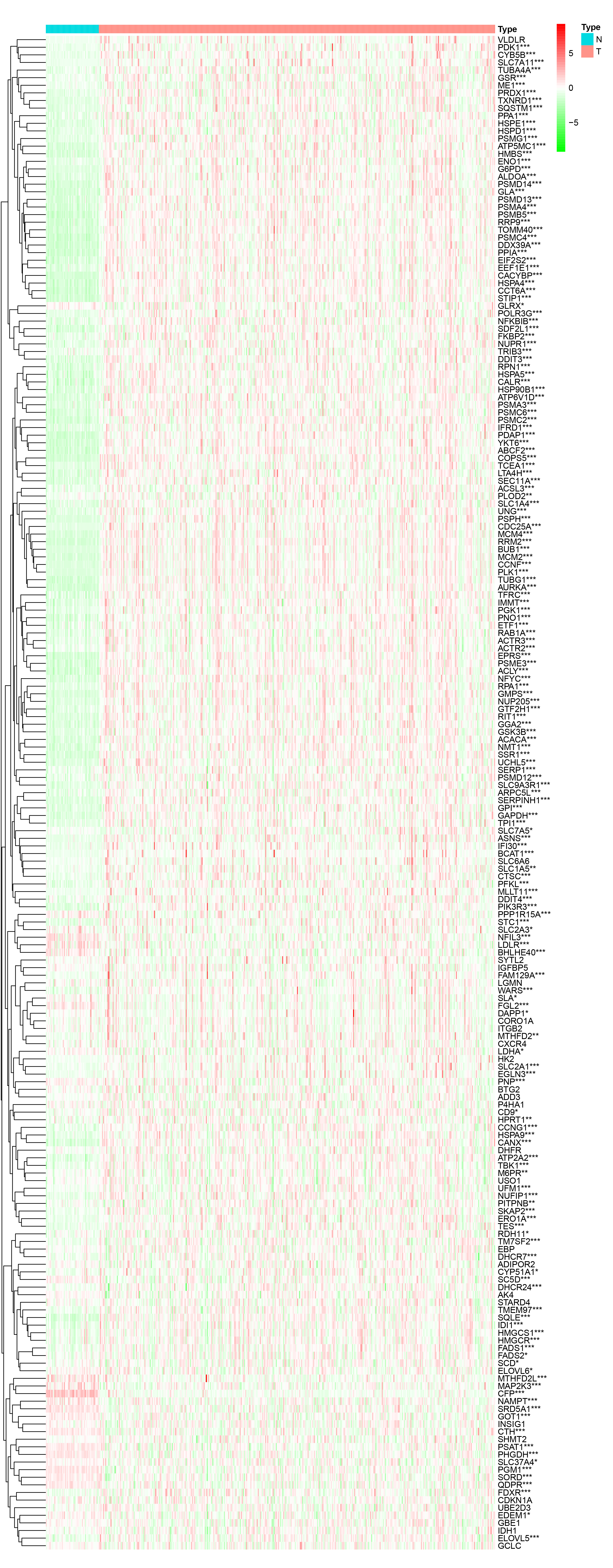

Supplement: Supplementary Materials — Supplementary file 1: the gene expression matrix of 199 mTORC1-related genes (TXT 890 kb). Supplementary file 2: the heatmap of 199 mTORC1-related genes (JPG 10489 kb). Supplementary file 3: the volcano plot of 199 mTORC1-related genes (JPG 571 kb). Supplementary file 4: the results of univariate Cox regression analysis for 160 genes (DOC 26.2 kb). Supplementary file 5: the results of multivariate Cox regression analysis for 101 genes (DOC 22.7 kb). Supplementary file 6: the results of decision curve analysis for three different models (JPG 988 kb). Supplementary file 7: the nomogram and calibration plots based on GEO cohort (JPG 608 kb). Supplementary file 8: the nomogram and calibration plots based on ICGC cohort (JPG 653 kb). [file 8291036.f1.zip › 8291036.f1/Supplementary file 2.jpg]

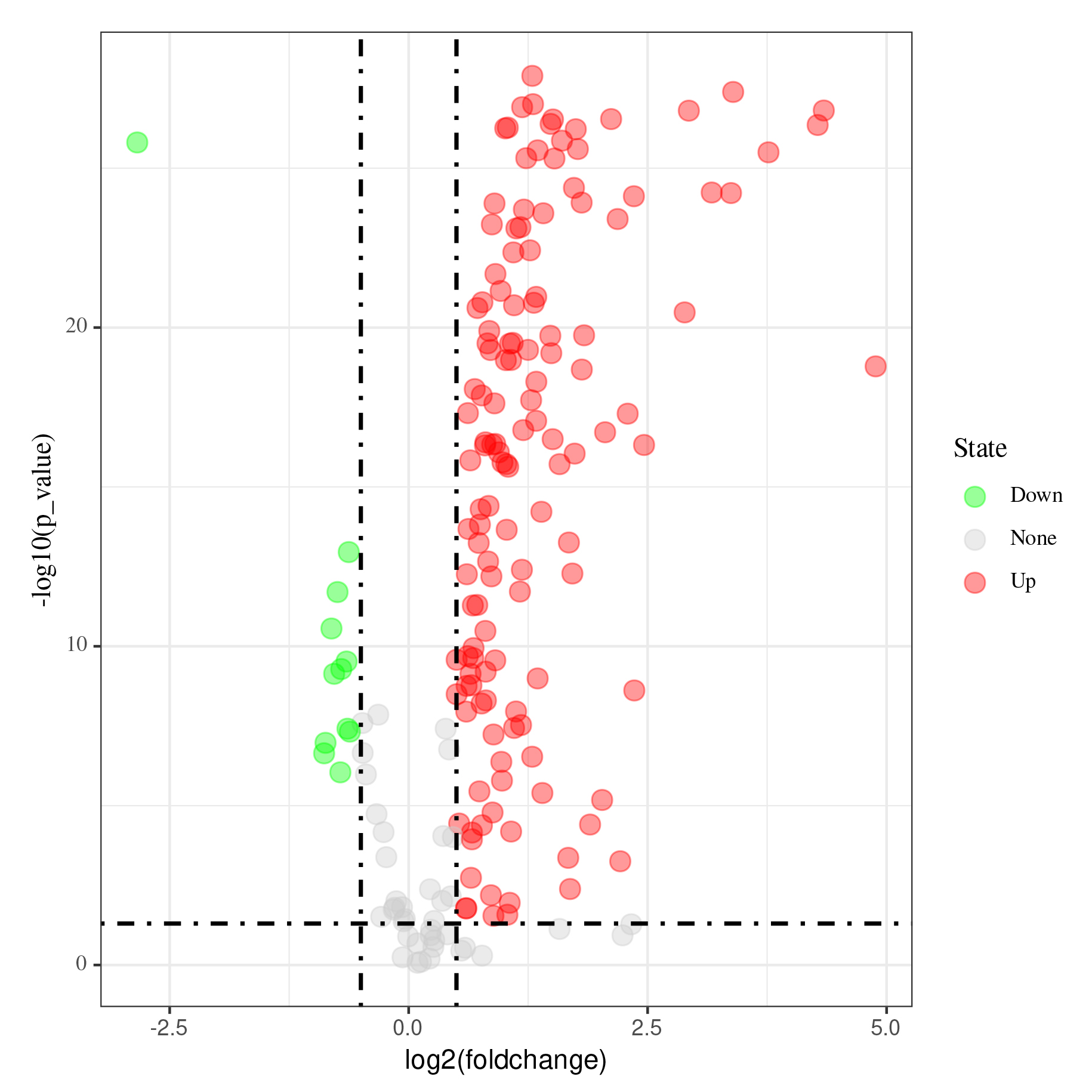

Supplement: Supplementary Materials — Supplementary file 1: the gene expression matrix of 199 mTORC1-related genes (TXT 890 kb). Supplementary file 2: the heatmap of 199 mTORC1-related genes (JPG 10489 kb). Supplementary file 3: the volcano plot of 199 mTORC1-related genes (JPG 571 kb). Supplementary file 4: the results of univariate Cox regression analysis for 160 genes (DOC 26.2 kb). Supplementary file 5: the results of multivariate Cox regression analysis for 101 genes (DOC 22.7 kb). Supplementary file 6: the results of decision curve analysis for three different models (JPG 988 kb). Supplementary file 7: the nomogram and calibration plots based on GEO cohort (JPG 608 kb). Supplementary file 8: the nomogram and calibration plots based on ICGC cohort (JPG 653 kb). [file 8291036.f1.zip › 8291036.f1/Supplementary file 3.jpg]

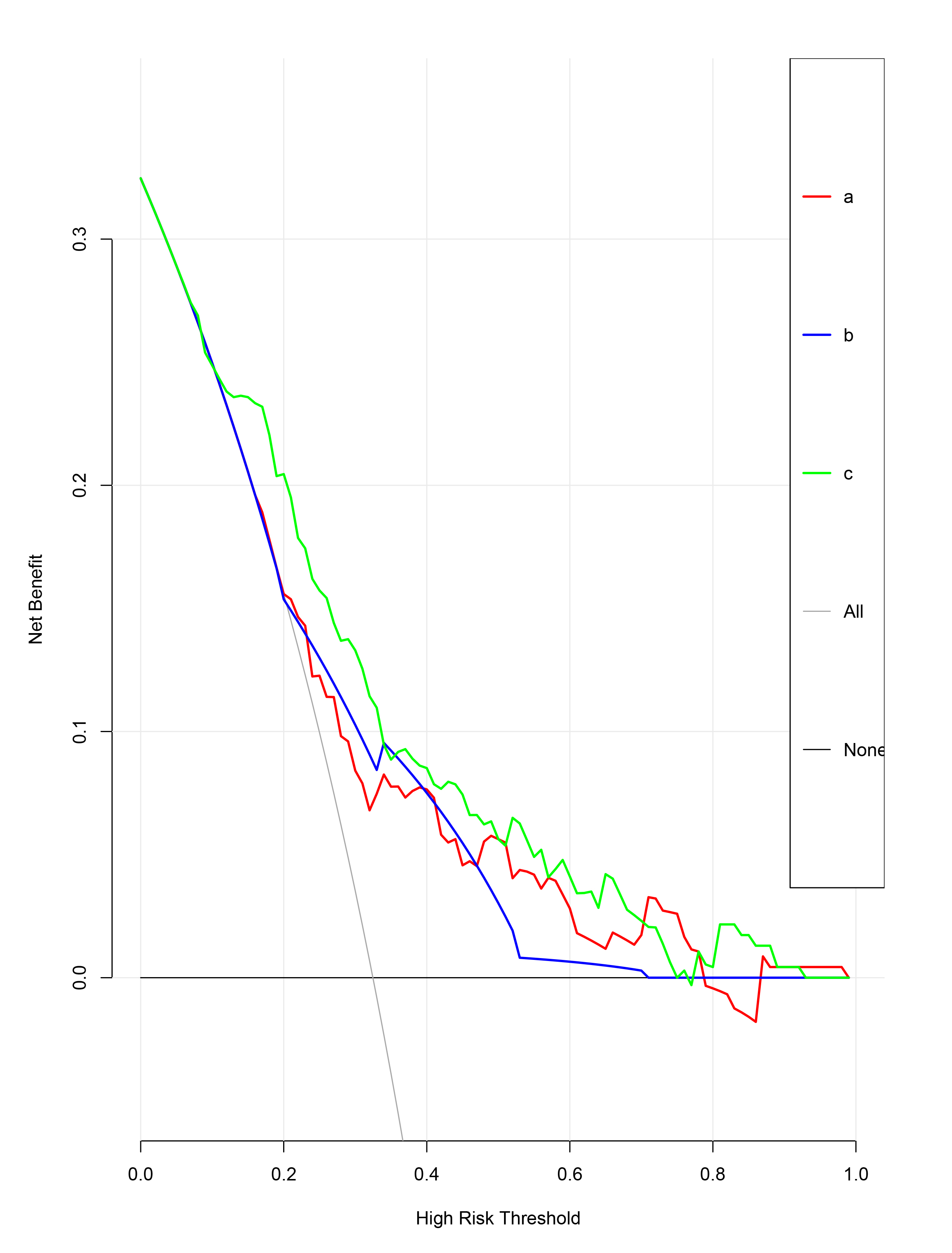

Supplement: Supplementary Materials — Supplementary file 1: the gene expression matrix of 199 mTORC1-related genes (TXT 890 kb). Supplementary file 2: the heatmap of 199 mTORC1-related genes (JPG 10489 kb). Supplementary file 3: the volcano plot of 199 mTORC1-related genes (JPG 571 kb). Supplementary file 4: the results of univariate Cox regression analysis for 160 genes (DOC 26.2 kb). Supplementary file 5: the results of multivariate Cox regression analysis for 101 genes (DOC 22.7 kb). Supplementary file 6: the results of decision curve analysis for three different models (JPG 988 kb). Supplementary file 7: the nomogram and calibration plots based on GEO cohort (JPG 608 kb). Supplementary file 8: the nomogram and calibration plots based on ICGC cohort (JPG 653 kb). [file 8291036.f1.zip › 8291036.f1/Supplementary file 6.jpg]

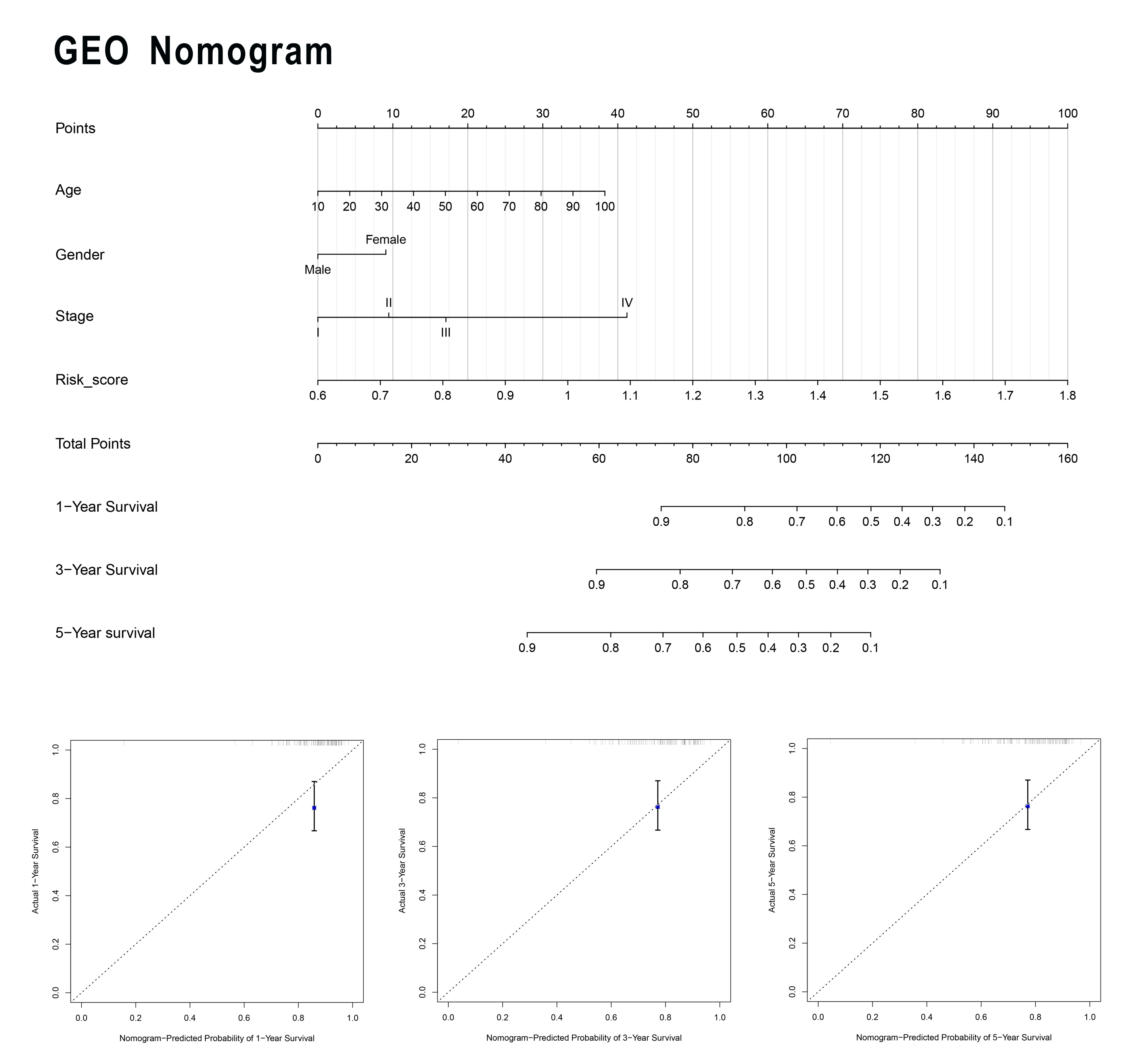

Supplement: Supplementary Materials — Supplementary file 1: the gene expression matrix of 199 mTORC1-related genes (TXT 890 kb). Supplementary file 2: the heatmap of 199 mTORC1-related genes (JPG 10489 kb). Supplementary file 3: the volcano plot of 199 mTORC1-related genes (JPG 571 kb). Supplementary file 4: the results of univariate Cox regression analysis for 160 genes (DOC 26.2 kb). Supplementary file 5: the results of multivariate Cox regression analysis for 101 genes (DOC 22.7 kb). Supplementary file 6: the results of decision curve analysis for three different models (JPG 988 kb). Supplementary file 7: the nomogram and calibration plots based on GEO cohort (JPG 608 kb). Supplementary file 8: the nomogram and calibration plots based on ICGC cohort (JPG 653 kb). [file 8291036.f1.zip › 8291036.f1/Supplementary file 7.jpg]

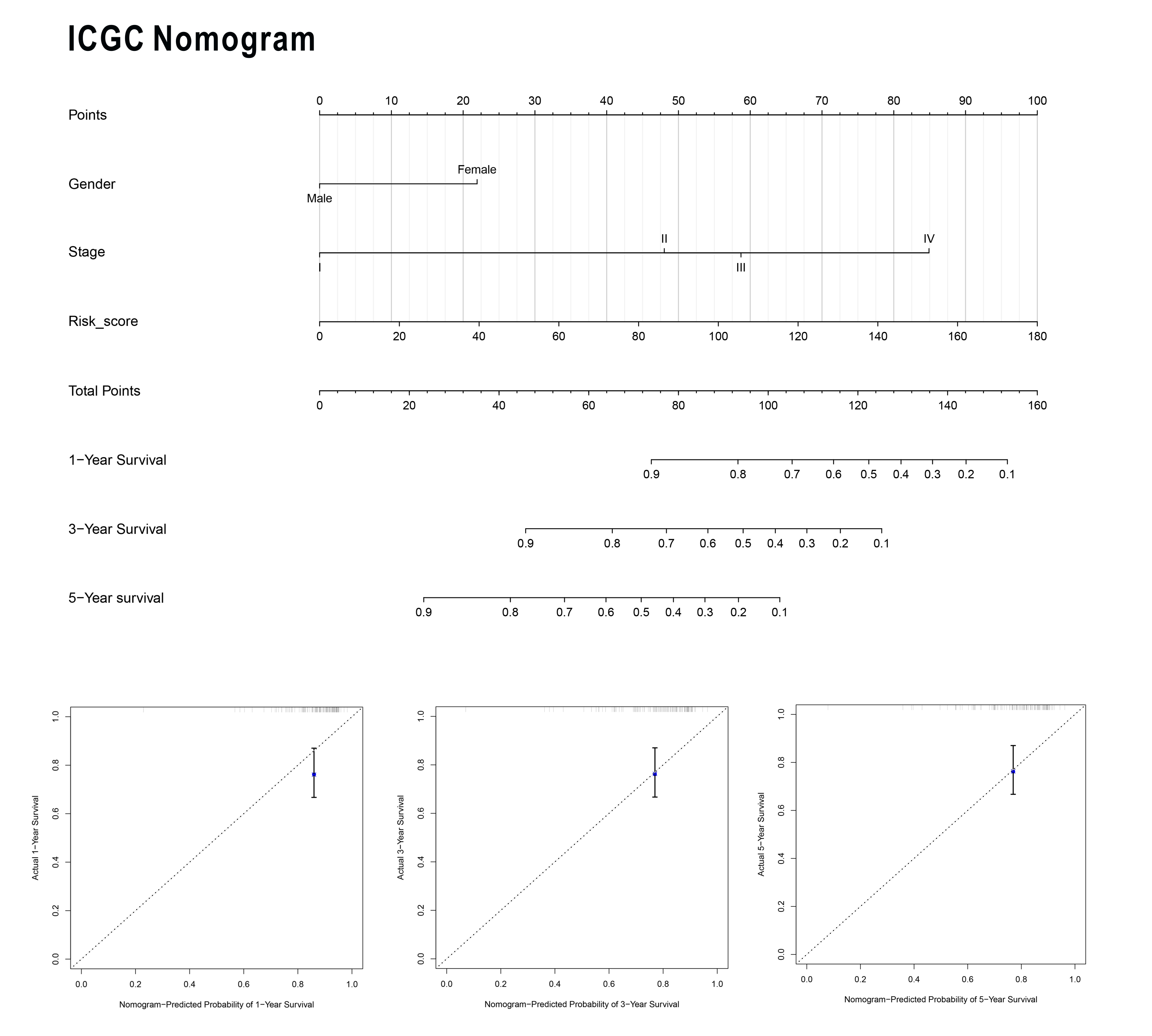

Supplement: Supplementary Materials — Supplementary file 1: the gene expression matrix of 199 mTORC1-related genes (TXT 890 kb). Supplementary file 2: the heatmap of 199 mTORC1-related genes (JPG 10489 kb). Supplementary file 3: the volcano plot of 199 mTORC1-related genes (JPG 571 kb). Supplementary file 4: the results of univariate Cox regression analysis for 160 genes (DOC 26.2 kb). Supplementary file 5: the results of multivariate Cox regression analysis for 101 genes (DOC 22.7 kb). Supplementary file 6: the results of decision curve analysis for three different models (JPG 988 kb). Supplementary file 7: the nomogram and calibration plots based on GEO cohort (JPG 608 kb). Supplementary file 8: the nomogram and calibration plots based on ICGC cohort (JPG 653 kb). [file 8291036.f1.zip › 8291036.f1/Supplementary file 8.jpg]
